# Redefining the Use of Digital Communities: AD Knowledge in an Online Educated Cohort of Midlife and Older Blacks

**DOI:** 10.1093/geroni/igab046.373

**Published:** 2021-12-17

**Authors:** Travonia Brown-Hughes, Alyssa Gamaldo, Corinne Pettigrew, Allison Caban-Holt, Nihal Mohamed, Roland Thorp, Jr

**Affiliations:** 1 School of Pharmacy, Hampton, Virginia, United States; 2 Penn State University, University Park, Pennsylvania, United States; 3 Johns Hopkins University, Baltimore, Maryland, United States; 4 Maya Angelou Center for Health Equity, Winston-Salem, North Carolina, United States; 5 Icahn School of Medicine at Mount Sinai Department of Oncological Sciences, New York, New York, United States; 6 Johns Hopkins Bloomberg School of Public Health, Baltimore, Maryland, United States

## Abstract

The normalization of memory loss continues to contribute to diagnostic delays among older adult African Americans with dementia. We utilized an innovative recruitment method to establish a solely online study to examine perceptions and knowledge levels of Alzheimer’s Disease in a highly educated geographically diverse cohort of 223 African Americans aged 50-84. Participants were recruited through largely electronic communications. Sample participants were primarily female (n=196), with 51.1% having completed a master’s degree, and 58.2% of participants with household incomes of $90,000 or higher. Study findings revealed that although highly educated, 42% of sample participants believed significant memory loss was a normal part of aging and 59.6% felt that God’s Will was a possible cause of AD. A sizable majority of participants, 86.5%, felt most family physicians were not trained to diagnose AD. Findings underscore the need for physician and community education within diverse populations, regardless of education and SES status.

